# A Prospective Randomized Study of Intensity-Modulated Radiation Therapy Versus Three-Dimensional Conformal Radiation Therapy With Concurrent Chemotherapy in Locally Advanced Carcinoma Cervix

**DOI:** 10.7759/cureus.21000

**Published:** 2022-01-07

**Authors:** Nidhi Sharma, Manoj Gupta, Deepa Joseph, Sweety Gupta, Rajesh Pasricha, Rachit Ahuja, Ajay S Krishnan, Aathira T S, Sagar Raut, Debanjan Sikdar

**Affiliations:** 1 Radiation Oncology, All India Institute of Medical Sciences, Rishikesh, Rishikesh, IND; 2 Radiation Oncology, All India Institute of Medical Sciences, Bhopal, Bhopal, IND; 3 Radiation Oncology, Vardhman Mahavir Medical College and Safdarjung Hospital, New Delhi, IND; 4 Radiation Oncology, All India Institute of Medical Sciences, New Delhi, New Delhi, IND

**Keywords:** acute toxicity, locoregional control, intensity modulated radiotherapy, 3dimensional-conformal radiotherapy, locally advanced carcinoma cervix

## Abstract

Background: External Beam Radiotherapy is the treatment of choice of locally advanced carcinoma cervix (LACC). The two techniques, three-dimensional conformal radiotherapy (3DCRT) and intensity-modulated radiotherapy (IMRT), have been compared previously in terms of outcomes and toxicities. IMRT has still not shown any benefit over 3DCRT in terms of local control and survival. Hence, the present study was conducted to compare local control and toxicities among both techniques.

Material & Methods: Fifty-four patients of LACC (FIGO IB2-IVA) were randomized to receive 50 Gray in 25 fractions by either 3DCRT or IMRT with concurrent cisplatin-based chemotherapy followed by brachytherapy. Plans were compared for planning target volume (PTV) coverage, dose to organs at risk (OAR), homogeneity index (HI), and conformity index (CI). Patients were assessed for acute toxicity and local control for three months.

Results: Out of 54 patients, 27 received treatment by 3DCRT and 27 by IMRT technique. Dosimetric evaluation for PTV coverage was similar in both arms. D15, D35, and D50 (dose to 15%, 35%, and 50% volume, respectively) for bladder were significantly reduced in the IMRT arm. Dosimetry for rectum and bowel bag was similar in both. There was a significantly decreased dose to femoral heads in the IMRT arm. Patients in the 3DCRT arm had significant grade 1 and 2 anemia and neutropenia compared to the IMRT arm. Local control for three months was similar in both the arms.

Conclusion: IMRT is associated with decreased acute hematological toxicity compared to 3DCRT with similar local control. Long-term follow-up is needed to assess any difference in long-term toxicity and survival between the two arms.

## Introduction

Cervical cancer remains the most common gynecologic cancer worldwide, with >500,000 women with cervical carcinoma and 233,000 women dying of the disease annually [1]. Sexually transmitted persistent human papillomavirus infection is the most important risk factor.

Locally advanced cancers (stages IB2-IVA) are managed with combined chemotherapy and radiotherapy [2]. External beam radiotherapy (EBRT) with concurrent chemotherapy and intracavitary brachytherapy remains the mainstay treatment for locally advanced carcinoma cervix (LACC). EBRT can be delivered using two-dimensional radiotherapy (2DRT), three-dimensional conformal radiotherapy (3DCRT), or intensity-modulated radiotherapy (IMRT). IMRT is associated with more accurate dose distributions and reduced dose to organs at risk (OARs), thus reducing side effects to the rectum, bladder, small bowel, and pelvic bones [3-6].

In intact cervical carcinoma, the bladder, rectum, and bowel are dynamic, and mobile structures in the pelvis directly proximal to the target structures. The dose distribution of bladder and rectal filling can vary based on these normal structures and alter the gross tumor volume (GTV) position [7,8]. Hence, the internal target motion and variability pose a great challenge in planning target margins, resulting in the role of IMRT in an intact cervix setting.

Various clinical trials [9-17] have been conducted worldwide to evaluate the most preferred treatment by comparing various clinical parameters, such as the chance of local and regional recurrence, radiation dose to normal structures (OARs), magnitude of side effects, and overall survival. With the available literature to date, we cannot conclusively say that IMRT is better than 3DCRT in all settings and among all patients. Hence, the current study was conducted to compare dosimetry parameters, acute toxicity, and local control between the two techniques.

## Materials and methods

This prospective, open-label randomized study was conducted between April 2018 and October 2019. Newly diagnosed biopsy-proven locally advanced cervical carcinoma based on the International Federation of Gynecology and Obstetrics (FIGO 2009) stage IB2-IVA was the selection criterion for the study. The study was approved by the Institute Ethics Committee. Informed consent was taken from all patients. Inclusion criteria were as follows: newly diagnosed biopsy-proven cervical carcinoma, hemoglobin of ≥10 g%, pre-treatment white blood cell (WBC) counts of >4,000/cu mm, platelet count of >100,000/cu mm, normal renal and liver functions, and Eastern Cooperation Oncology Group (ECOG) performance status of ≤2. Postoperative patients with cervical carcinoma, who underwent any previous treatments for pelvic cancers, and with synchronous malignancy were excluded from the study. Detailed history and physical examination were conducted for all patients. Pretreatment complete hemogram (hemoglobin, WBC count, and platelet count), liver function tests, renal function tests were performed. All patients underwent contrast-enhanced CT of the whole abdomen and pelvis and chest x-ray. Cystoscopy and procto-sigmoidoscopy were also performed whenever clinically indicated.

**Figure 1:**
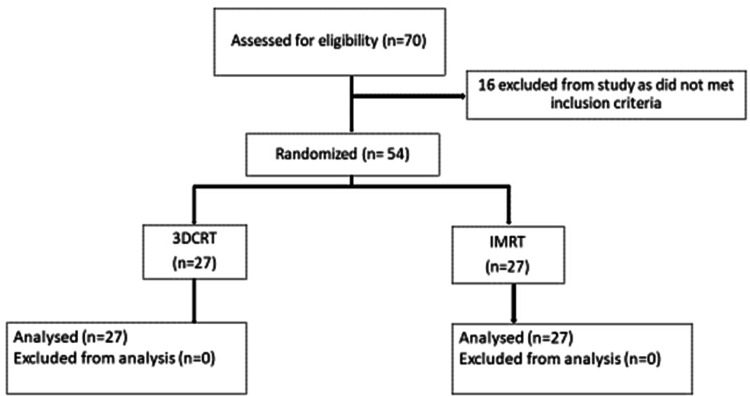
Flow diagram of the study

Computer-based randomization was performed into either the 3DCRT or IMRT arm. Patients in both arms received EBRT 50 Gy in 25 fractions for >5 weeks with weekly concurrent cisplatin 35 mg/m^2^, followed by high-dose-rate intracavitary brachytherapy (ICBT), 7 Gy to point A in three once-weekly sessions. A flow diagram of the study is summarized in Figure 1.

Immobilization and simulation

The simulation was performed in a supine position and immobilized using a 4-clamp pelvic thermoplastic mask. Standard bladder protocol was maintained for all patients during simulation and treatment. At the beginning of the simulation, patients were instructed to empty their bladder and then take 1 L of water for one hour and were advised not to void in between. Contrast-enhanced CT simulation was performed at the end of one hour. A vaginal marker was placed at the lower extent of the cervical disease during CT. Planning images were then generated by CT scanning. The three orthogonal lasers were used to establish the patient coordinate system. The crosswire points in X, Y, and Z planes were marked on patients with lead wires (fiducial markers). A 2.5-mm slice thickness was obtained from L2 to mid-thigh for all planning CT images.

Contouring

The CT image dataset obtained was then imported to the treatment planning system (MONACO, Elekta). These images became the basis for contouring the GTV, clinical target volume, planning target volume (PTV), and OAR, such as the bladder, rectum, bowel bag, and femoral heads. Delineation of the target volumes and OARs was performed based on consensus guidelines of the radiation therapy oncology group [18].

Planning

All planning was performed on the MONACO Treatment Planning System. Target planning constraints used were PTV D95 of >95% of the prescribed dose (PD), bladder V40 Gy of <75%, rectum V40 Gy of <85%, femoral heads Dmax of ≤50 Gy, and bowel V45 of ≤195 mL.

3DCRT planning

**Figure 2:**
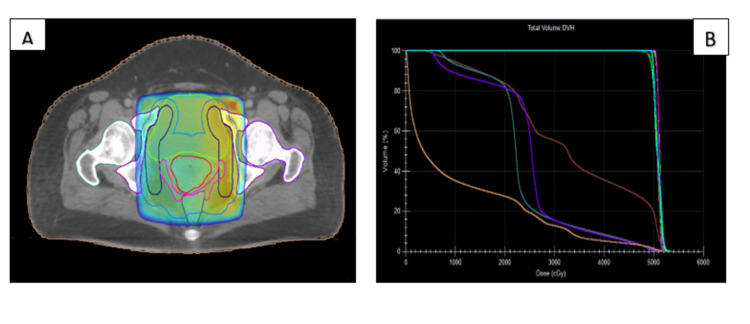
Target volume coverage (A) and dose-volume histogram (B) for a 3DCRT plan

Four fields (AP-PA and two lateral fields) with a couch angle of 0° were used in all 3DCRT plans (Figure 2 A). The borders were: Superior - L5/S1; Inferior - bottom of the obturator foramen or 3 cm below vaginal marker; Lateral - 2 cm on the pelvic brim; Anterior - 5 mm anterior to pubic symphysis; and Posterior - S2/S3. The isocenter was placed at the geometrical center of the PTV. To manage hot/cold spot and dose homogeneity to optimize PTV coverage, the weight of particular fields was decreased/increased by changing the monitoring units, while minimizing exposure of the urinary bladder and rectum. Beam energy used was 6/15 MV. Plans were calculated in the Monaco Environment 5.11.02 using Monte Carlo Analytical Algorithm with a dose matrix resolution of 3 mm.

IMRT planning

**Figure 3:**
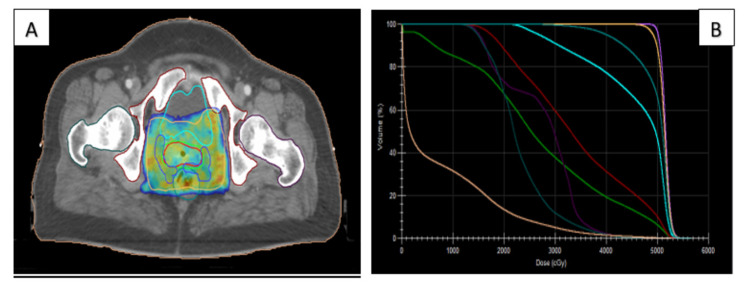
Target volume coverage (A) and dose-volume histogram (B) for an IMRT plan

IMRT plans were performed using seven to nine beams (Figure 3A). The isocenter was placed at the geometrical center of the PTV. Plans were optimized in the Monaco Environment 5.11.02, and dose calculation was performed using the Monte Carlo Analytical Algorithm with a dose matrix resolution of 3 mm.

Plan evaluation

Each plan was evaluated by a radiation oncologist for target coverage and dose to OARs (Figures 2B, 3B). For the target coverage, the parameters evaluated were D95 and D99 (dose to 95% and 99% PTV, respectively), Dmax (Maximum dose) defined as the dose received by PTV of not >107% of the PD, and Dmin (minimum dose) defined as the dose received not <93% of the PD. Conformity index (CI) and homogeneity index (HI) and for 95% of the PTV was calculated for both techniques using the following formulas:

Conformity Index = Volume receiving 95% of prescribed dose/Planning Target Volume

Homogeneity Index = Dose received in 95% of PTV volume/ dose received in 5% of PTV volume

The OAR dose was compared using the following parameters:

For the bladder and rectum, dosimetric parameters were described as D15, D35, and D50 (dose to 15%, 35%, and 50% of the organ volume). The dosimetric parameter assessed in the femoral heads was the maximum dose received (Dmax) of 0.03 mL volume. The parameters of the bowel bag (small and large intestines) were described in volumes of V45 Gy (volume in mL receiving 45 Gy).

 

Treatment

Patients were treated on Elekta Versa-HD Linear Accelerator. Before each treatment, the bladder protocol was followed for all patients. Patients treated with both 3DCRT and IMRT underwent cone-beam CT (X-Ray Volumetric Imaging- XVI) imaging to verify the treatment setup.

Concurrent chemotherapy

Patients were administered Inj cisplatin (35 mg/m^2^) on a weekly basis during EBRT not exceeding 50 mg. All patients were adequately hydrated with 2-2.5 L of intravenous fluids and supplemented with an injection of KCl and MgSO_4_ during chemotherapy. Radiotherapy was delivered within one hour of cisplatin administration.

Brachytherapy

This was followed by three applications of intracavitary brachytherapy of 7 Gy for each fraction one week apart to point A.

Toxicity assessment and follow-up

Weekly clinical examination and CBC and KFT were performed to assess chemo radiation-induced acute toxicities.

Toxicity within 90 days of starting radiotherapy was considered for acute toxicity and Common Terminology Criteria for Adverse Events version 4 (CTCAE v4) for grading toxicities. Patients were followed up monthly up to three-month post-completion of radiation treatment. During each follow-up, the response evaluation was performed by clinical assessment. The three-month response evaluation was performed clinicoradiologically (CECT Whole abdomen and pelvis) using the RECIST 1.1 criteria.

Statistical analysis

The statistically significant difference between each set of dosimetric parameters was determined using the Student’s t-test. A chi-square test/Fisher’s exact test was used to compare toxicity between arms. A p-value of <0.05 was considered significant.

## Results

Patient characteristics

A total of 54 patients with cervical carcinoma (stage IB2-IVA) were enrolled in the study. Patient characteristics are described in Table 1. No statistically significant difference was observed between the two arms in terms of patient characteristics.

**Table 1:** Comparison of patient characteristics in both arms 3DCRT, three-dimensional conformal radiotherapy; IMRT, intensity-modulated radiotherapy; p/v, per vaginum; SCC, squamous cell carcinoma;  HPE, histopathological examination

Characteristics	3DCRT	IMRT	P-value
Age			
Mean (years)	55.2 ± 10.51	56.8 ± 10.25	0.458
Median (years)	58	57	0.178
Comorbidities			
Hypertension	3 (11.1%)	3 (11.1%)	0.228
Diabetes	3 (11.1%)	0	
Hepatitis B	1 (3.7%)	0	
Hepatitis C	1 (3.7%)	0	
Tuberculosis	1 (3.7%)	0	
Addictions			
Smoking	7 (25.9%)	6 (22.2%)	0.949
Tobacco chewer	2 (7.4%)	2 (7.4%)	
Chief complaints			
White discharge p/v	20 (74.1%)	21 (77.7%)	0.170
Bleeding p/v	17 (62.9%)	13 (48.1%)	
Pain abdomen	11 (40.7%)	16 (59.2%)	
Low back ache	6 (22.2%)	1 (3.7%)	
ECOG			
0	11 (40.7%)	10 (37%)	0.597
1	11 (40.7%)	13 (48%)	
2	5 (18.6%)	4 (15%)	
Menopausal status			
Post-menopause	21 (77.8%)	21 (77.8%)	0.949
Pre-menopause	6 (22.2%)	6 (22.2%)	
Parity			
Multiparous	27 (100%)	25 (92.6%)	0.170
Nulliparous	0	2 (7.4%)	
HPE			
Keratinising SCC	15 (55.6%)	17 (63%)	0.540
Non-Keratinising SCC	11 (40.7%)	10 (37%)	
Adenocarcinoma	1 (3.7%)	0	
Stage			
IB2	2 (7.4%)	1 (3.7%)	0.570
IIA	0 (0%)	0 (0%)	
IIB	12 (44.5%)	17 (63%)	
IIIA	1 (3.7%)	2 (7.4%)	
IIIB	10 (37%)	6 (22.2%)	
IVA	2 (7.4%)	1 (3.7%)	

Dosimetric results

The PTV coverage was similar between both arms. Within the target dose distribution showed a similar HI and CI for the two arms. Dosimetric details for PTV coverage are shown in Table 2.

**Table 2:** Mean dosimetric parameters for PTV coverage of the two arms Dx (% of PD), % of PD to X % of PTV; Dmax, maximum dose in % of PD; Dmin, minimum dose in % of PD; HI, homogeneity index; CI, conformity index; IMRT= intensity‑modulated radiotherapy; 3DCRT= three‑dimensional conformal radiotherapy

Dosimetric parameters	3DCRT	IMRT	P-value
PTV			
D99	47.80 ± 0.90	47.66 ± 0.92	0.59
D95	48.91 ± 0.74	48.83 ± 0.84	0.872
Dmax	53.69 ± 0.90	53.39 ± 1.16	0.715
Dmin	48.83 ± 2.0	49.31 ± 2.21	0.378
HI	0.93 ±0.03	0.95 ±0.02	0.274
CI	0.98 ± 0.01	0.98 ± 0.01	0.786

The mean values of D15, D35, and D50 of the urinary bladder were lower in IMRT as compared to the 3DCRT arm. Dose to the rectum was marginally lower with IMRT as compared to 3DCRT but results were non-significant. The bowel bag volume receiving 45 Gy was similar in both arms. The Dmax received in the femoral head was higher in the 3DCRT arm as compared to the IMRT arm. The dose details to OAR are mentioned in Table 3.

**Table 3:** Mean dosimetric parameters for organs at risk in the two arms Dx, Dose to x% of volume; V45, volume receiving 45 Gy; Dmax, maximum dose; IMRT, intensity‑modulated radiotherapy; 3DCRT, three‑dimensional conformal radiotherapy

Dosimetric parameters	3DCRT	IMRT	P-value
Urinary Bladder			
D15 (Gy)	51.17 ± 0.44	50.8 ± 0.97	0.003
D35 (Gy)	50.72 ± 0.96	49.8 ±1.29	0.009
D50 (Gy)	49.93 ±1.76	48.90 ± 2.24	0.023
Rectum			
D15 (Gy)	51.32 ± 1.0	50.94 ± 0.92	0.165
D35 (Gy)	50.68 ± 0.88	49.88 ±2.29	0.165
D50 (Gy)	50.24 ±1.06	48.88± 1.65	0.105
Bowel Bag V45 (cc)	486.92 ± 183.78	415.07 ± 110.21	0.242
Femoral Head Dmax (Gy)			
Right	50.28 ± 1.6	48.41 ± 1.8	0.057
Left	50.23 ± 2.14	47.68 ± 1.87	0.042

Toxicity profile

No significant difference was observed in acute toxicities of genitourinary and gastrointestinal toxicities in both arms. However, the number of patients with grade 1 anemia and neutropenia was higher in the 3DCRT than in the IMRT arm as shown in Table 4.

**Table 4:** Acute toxicities in both arms GU, genitourinary; GI, gastrointestinal; IMRT, intensity‑modulated radiotherapy; 3DCRT, three‑dimensional conformal radiotherapy

Acute toxicities	Grade	3DCRT	IMRT	P-value
GU toxicity	1	13	11	0.472
	2	1	0	
	≥3	0	0	
GI toxicity	1	10	11	0.960
	2	1	1	
	≥3	0	0	
Anemia	1	10	7	0.034
	2	2	2	
	≥3	0	0	
Neutropenia	1	15	8	0.039
	2	1	1	
	≥3	0	1	
Thrombocytopenia	1	4	2	0.385
	2	0	0	
	≥3	0	1	

Early clinical outcome

At the three-month follow-up, the clinical and radiological response assessment showed complete response in 42 (77.7%) patients (21 in 3DCRT and 21 in IMRT arm), and eight (15%) patients had a partial response (four in each arm). In the 3DCRT arm, one (1.8%) patient developed bone metastasis. In the IMRT arm, one (1.8%) patient died due to urosepsis and two (3.7%) developed metastasis (in the lung and vulva).

## Discussion

Naik et al. [9] aimed to achieve the target coverage by both techniques. However, IMRT plans had significantly better PTV coverage and CI than that in the 3DCRT. In our study, the target coverage was similar in both 3DCRT and IMRT arms in terms of D99, D95, Dmax, and Dmin. Further, CI and HI were similar in both treatment arms.

Naik et al. [9] investigated the bowel volumes irradiated in patients with cervical carcinoma treated with 3DCRT and IMRT and observed that the means of V45 in the bowel bag in 3DCRT and IMRT arms were 227 mL and 132 mL, respectively. Moreover, they concluded that rectal parameters D15, D35, and D50 of the IMRT arm were significantly lower than that of the 3DCRT arm. Fumaiki et al. [10] compared IMRT and 3DCRT in the cervical carcinoma with concurrent chemotherapy and observed that the V45 of the bowel bag in the IMRT arm was 485 mL, whereas that of the 3DCRT arm was 891 mL, a significant reduction and also statistically significant. Gandhi et al. [19] studied gastrointestinal toxicities in patients with cervical carcinoma treated with 3DCRT and IMRT and observed a significant reduction in both grade II and III toxicities of acute and late gastrointestinal toxicities in patients treated with IMRT than those treated with 3DCRT.

The current study demonstrated the bowel volume of 45 Gy was <490 mL in both arms 485 mL in 3DCRT and 414 mL in the IMRT arm, which is different from existing reports. Furthermore, this study showed reduced D15, D35, and D50 of the rectum in the IMRT arm compared to the 3DCRT arm; however, our results were statistically non-significant. Patients with IMRT had similar grade I and fewer grade II acute bowel toxicities than patients with 3DCRT.

Mundt et al. [20] compared 3DCRT and IMRT in cervical carcinoma and found that grade 2 genitourinary toxicities reduced from 50% to 11% in the IMRT arm compared to the 3DCRT arm. Naik et al. [9] showed that IMRT offered a significant advantage over 3DCRT in terms of reduced OAR dose, especially the doses received by the bladder in 2.09% (D15), 14.623% (D35), and 32.57% (D50).

We observed a significant reduction in bladder volumes D15, D35, and D50 irradiated with IMRT when compared to 3DCRT, which is similar to Naik et al.’s findings. Moreover, patients treated with IMRT had fewer grade I and II acute bladder toxicities, although non-significant than those treated with 3DCRT, which was in concordance with Mundt et al. and Naik et al.’s studies. We did not observe any grade III/IV genitourinary toxicities in both arms.

Erpolat et al. [21] compared the clinical treatment effects of 3DCRT in cervical carcinoma with IMRT, and local control rates were similar in both techniques.

We observed that the complete response rates at three months after treatment completion were 74% and 77% in the 3DCRT and IMRT arms, respectively, slightly less than those observed by Erpolat et al. [21] and Naik et al. [9]. Inferior results in the current study may be due to higher stage of patients and associated comorbidities. Long-term follow-up is required to assess the locoregional control.

Our results showed that patients treated with either of conformal techniques of radiotherapy, 3DCRT, and IMRT had almost equal locoregional disease control at three months. When toxicity profiles were compared, fewer grade I and II hematological toxicities were observed in the IMRT arm. We did not observe any grade III or IV toxicities in both arms. Although the bladder received a significantly lesser dose in IMRT plans compared to those in 3DCRT, this was not reflected based on the difference in the acute toxicity between the two arms. The IMRT arm was associated with lesser hematological toxicities, such as anemia and neutropenia, which can be attributed to patient-related factors like the general condition and compliance; tumor-related factors like normal tissue repair; and treatment-related factors such as daily reproducibility, internal organ motion (both inter fractional, intrafractional, rectal, and bladder filling). Therefore, more accurate and potential predictive parameters should be identified.

## Conclusions

Based on our study, we can conclude that in low-resource settings, 3DCRT may be a good treatment option for patients with LACC but IMRT can also be effectively used to reduce the irradiated volume of the normal tissues with a better side effect profile.

Our study is limited by its short follow-up time and relatively small sample size. Long-term follow-up is required to assess any survival difference or long-term toxicity between the two study arms.
